# Long-Term Efficacy, Visual Performance and Patient Reported Outcomes with a Trifocal Intraocular Lens: A Six-Year Follow-up

**DOI:** 10.3390/jcm10092009

**Published:** 2021-05-07

**Authors:** Joaquín Fernández, Manuel Rodríguez-Vallejo, Javier Martínez, Noemi Burguera, David P. Piñero

**Affiliations:** 1 Qvision, Department of Ophthalmology of VITHAS Almería Hospital, 04120 Almería, Spain; joaquinfernandezoft@qvision.es (J.F.); javiermartinezopt@qvision.es (J.M.); noemiburgueraid@qvision.es (N.B.); 2 Department of Optics, Pharmacology and Anatomy, University of Alicante, 03009 Alicante, Spain; david.pinyero@ua.es; 3 Department of Ophthalmology (IMQO-Oftalmar), Vithas Medimar International Hospital, 03016 Alicante, Spain

**Keywords:** multifocal intraocular lens, long-term, safety, efficacy, defocus curve, light distortion

## Abstract

(1) Background: To evaluate the efficacy at 6 years postoperative after the implantation of a trifocal intraocular lens (IOL) AT Lisa Tri 839MP. The secondary objective was to evaluate the contrast sensitivity defocus curve (CSDC), light distortion analysis (LDA), and patient reported outcomes (PROs). (2) Methods: Sixty-two subjects participated in phone call interviews to collect data regarding a visual function questionnaire (VF-14), a patient reported spectacle independence questionnaire (PRSIQ), and questions related to satisfaction and decision to be implanted with the same IOL. Thirty-seven of these subjects were consecutively invited to a study visit for measurement of their visual acuity (VA), CSDC, and LDA. (3) Results: The mean monocular distance corrected VA was −0.05, 0.08, and 0.05 logMAR at far and distances of 67 cm and 40 cm, respectively. These VAs were significantly superior to those reported in previous literature (*p* < 0.05). The total area under the CSDC was 2.29 logCS/m^−1^ and the light distortion index 18.82%. The mean VF-14 score was 94.73, with 19.4% of subjects requiring spectacles occasionally for near distances, and 88.9% considering the decision of being operated again; (4) Conclusions: Long-term AT LISA Tri 839MP IOL efficacy results were equal or better than those reported 12 months postoperatively in previous studies. The spectacle independence and satisfaction rates were comparable to those reported in short-term studies.

## 1. Introduction

The intraocular lens (IOL) AT LISA tri 839MP (henceforth referred to as 839MP; Carl Zeiss Meditec AG, Germany) was, together with the FineVision (Physiol S.A., Liege, Belgium), the first trifocal IOL that received the CE mark in 2012 and 2010, respectively [1]. The short-term safety and efficacy of 839MP has been widely reported (1 to 6 month follow-up) [2,3,4]. Long-term studies with 839MP are commonly referred to as those that involve 1 or 2 year follow-up periods [5,6,7,8,9]. To the best of our knowledge, there is only one retrospective study evaluating a longer period of 3–4 years follow-up, in which only Nd-YAG rates were analyzed [10]. The 839MP IOL remains one of the most popular and frequently implanted multifocal IOL (MIOL), and thus, the results for longer periods are considered to be of great interest. The main aim of this study was to evaluate the efficacy 6 years after binocular implantation of the 839MP IOL. The secondary aim was to evaluate the contrast sensitivity defocus curves, light distortion analysis, and patient reported outcomes (PROs) in eyes implanted with this modality of trifocal IOL.

## 2. Materials and Methods

This retrospective and cross-sectional study was designed to be conducted during the COVID-19 pandemic period, following the recommendations of the European Medicines Agency [11]. To minimize the pandemic risks, the study consisted of two stages, namely: (1) PRO evaluations through a phone call for all of the patients implanted with 839MP IOL from March 2014 to June 2015, who met the inclusion/exclusion criteria, and (2) a visit to our center for visual performance measurements by the patients who were consequently selected during the phone call interviews. The study was approved by the Ethics Committee of Research, Almería Center, Torrecardenas Hospital Complex (QV-20-02), and was performed in accordance with the tenets of the Declaration of Helsinki. Oral consent was provided in stage 1 during the phone call, and the signed consent was provided in stage 2. The study was registered at ClinicalTrials.gov (registration number: NCT04861909).

### 2.1. Subjects

Ninety-two patients that underwent refractive lens exchange or cataract surgery were identified in our historical database to be contacted via phone for the assessment of long-term PROs (first stage). The inclusion criteria for this first stage were patients older than 45 years old at the time of surgery and younger than 80 years old at the time of the phone call interview; patients who had undergone surgery in the first eye at least 78 months prior to the interview, and 66 months at least in the second eye; and no reports of surgical complications recorded in the clinical history that could deteriorate visual acuity. The exclusion criteria were any ocular disease reported in the clinical history leading to a visual acuity loss, such as recurrent anterior segment diseases and retinal and optic nerve alterations, and any ocular surgery, including corneal laser refractive surgery, prior or after the surgery. A total of 77 patients who met the mentioned inclusion and exclusion criteria after reviewing the clinical history were attempted to be contacted by phone. However, only 62 subjects were successfully contacted, interviewed, and consecutively invited to visit our centre for a vision assessment to achieve the required sample size for the second stage (*n* = 37). Exclusion criteria for completing the vision assessment in the second stage were posterior capsular opacification (PCO) grade density ≥2 according to surgeon criteria that induced a visual loss in corrected distance visual acuity (CDVA) ≥ 0.2 logMAR and irregular corneal astigmatism at 4 mm above 0.5 µm measured with Pentacam HR (Oculus Optikgeräte GmbH, Wetzlar, Germany).

### 2.2. Clinical Procedures

The PROs collected during the phone call interview were the Spanish validated Visual Function Questionnaire (VF-14) without spectacles [12,13]; a non-validated Spanish translation of the English validated patient reported spectacle independence questionnaire (PRSIQ) [14]; and three independent questions about satisfaction with vision, the level of disturbance related to photic phenomena, and the decision of whether the patient would choose to be implanted again with the same MIOL, considering their vision without any spectacle correction.

Patients conducting the visual performance assessment visit underwent a slit-lamp and medical exploration for adverse event detection [15], including PCO density grading from 0 to 4 in the eyes without a previous Nd-YAG capsulotomy and anterior capsular opacification [16,17]. The best spectacle refraction was obtained at 4 m (−0.25 D added for correction to infinity) and monocular visual acuities, uncorrected and distance corrected, were obtained at far, intermediate (67 cm), and near vision (40 cm) using an iPad ETDRS chart (VisionC 2.0; https://www.test-eye.com/en/apps-for-eye-care-research/visionc; Accessed 5 May 2021, Spain) [15]. Binocular uncorrected visual acuities were also measured at the three distances mentioned. Standard notation was used for abbreviating the visual acuities [18].

An eye randomization (RandomIZE: Randomization Tool, IOS App) was performed, selecting only the right or left eye for some of the tests requiring longer testing times, as described below. Some retrospective data and biometric eye parameters described in Table 1 were also obtained for the eye randomly selected with the Pentacam AXL (Oculus Optikgeräte, Wetzlar, Germany), IOL Master 500 (Carl Zeiss Meditec AG, Jena, Germany), and Keratograph 5M systems (Oculus Optikgeräte, Wetzlar, Germany).

The same iPad was used to measure the contrast sensitivity defocus curve (CSDC) with a Multifocal Lens Analyzer (3.0; Snellen letters; https://www.defocuscurve.com/en/; Accessed 5 May 2021) [19,20]. Defocus lenses from +1.00 D to −4.00 D, in steps of +0.50 D, were selected for measuring CSDC at 4 m in the randomly selected eye, with a best distance spectacle refraction and vergence distance correction of +0.25 D. The Light Distortion Analyzer (CEORLab, University of Minho, Braga, Portugal) was used to measure the photic phenomena monocularly, with the best distance correction in the eye randomly selected and binocularly without correction 2 m from the observer (proximal vergence corrected with +0.50 D). The following three variables were obtained: the light distortion index (LDI, %), the best fit circle irregularity (BFCi, mm), and the best fit circle radius (BFCr, mm) [5].

### 2.3. Surgery and Intraocular Lens

All cataract surgeries were performed by the same surgeon (JF) by phacoemulsification, either assisted or not assisted by a femtosecond laser, at Qvision (Ophthalmology Department, Vithas Virgen del Mar Hospital). Temporal clear corneal incisions were conducted during surgery in all cases with the insertion of the IOLs at 0–180° location. The 839MP was the trifocal IOL implanted in all cases, which has been widely described in previous peer-reviewed literature [1,21].

### 2.4. Statistical Analysis

The primary end-points were the uncorrected and corrected monocular visual acuities at far, intermediate, and near distances. Secondary end-points were contrast sensitivity defocus curves, light distortion analysis, and patient reported outcomes. Only the randomized eye was considered for the inferential statistics analysis. In a non-inferiority design, with one sided testing, the null hypothesis of the mean differences equal or higher to the 12-month means reported by Mojzis et al. [6] for primary end-points plus a margin of 0.1 logMAR was planned before the study. The sample size was computed with the software G*Power (version 3.1; https://www.gpower.hhu.de/; Accessed 5 May 2021), considering the mean differences of 0.1 logMAR and the standard deviations reported by Mojzis et al. [6]. The sample size of 37 subjects was selected considering a type I error probability of 0.05 and a statistical power of 0.99 for the primary end-point. Normal distributions were tested with the Shapiro–Wilk test, and the Wilcoxon-signed rank test for one sample was selected for testing the hypothesis for median differences because of the non-normal distribution of variables. Descriptive statistics are detailed in the results section as mean ± standard deviation (median (interquartile range)). A survival analysis was performed for the assessment of the Nd:YAG rates. Correlations were assessed with Spearman rho, and associations between ordinal variables from the questionnaires were analyzed with Sommers’d. Likewise, a multiple linear regression analysis was performed to define the variables predicting the magnitude of LDI. The Refractive Analysis toolbox for MATLAB (R2019; MathWorks, Natick, MA, USA) was used for conducting the standard plots [22]. Shifted defocus curves were corrected before computing the descriptive statistics, and the areas under the defocus curve (AUCs) for total (T, +1 to −4 D), far (F, +0.5 to −0.5 D), intermediate (I, −0.5 to −2 D), and near (N, −2 to −4 D) ranges were calculated. SPSS version 24 software (SPSS Inc., Chicago, IL, USA) was used for the statistical analysis.

## 3. Results

### 3.1. Recruited Subjects

From the 92 subjects reviewed, 15 subjects were excluded for the following reasons: five had previous laser photorefractive keratectomy (PRK) interventions, four developed concomitant diseases, one had a surgical complication with a sulcus implant in one eye, two had additional surgical interventions during the follow-up, two cases reported of sporadic double vision, and one subject had a toric IOL implanted in one eye. Sixty-two subjects were finally interviewed, 26 men and 36 women, with mean age of 60.74 ± 5.92 (60 [9]) years old. From those subjects, a total of 37 consecutive patients, whose demographic variables are shown in Table 1, attended the study visit at our clinic—15 men and 22 women. None of the 37 consecutive patients invited to the study visit had to be excluded for not accomplishing the inclusion/exclusion criteria defined in the protocol.

### 3.2. Efficacy and Accuracy

**Figure 1:**
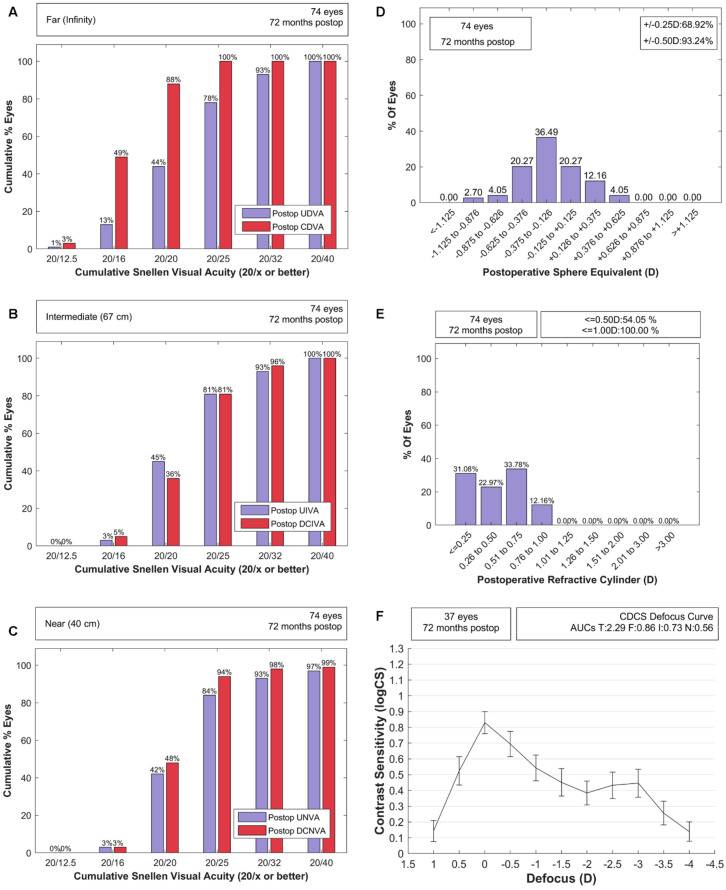
Efficacy plot for (**A**) far, (**B**) intermediate, and (**C**) near distances showing the cumulated percentage of eyes achieving a particular level of uncorrected and corrected visual acuity postoperatively. Percentage of eyes achieving (**D**) a postoperative sphere equivalent non-relative to the intended target and (**E**) a postoperative cylinder refraction. (**F**) Mean monocular contrast sensitivity defocus curve with 95% confidence interval in error bars and with areas under the curve (AUCs) for total (T), far (F), intermediate (I), and near ranges (N).

Monocular visual acuities for the randomized eyes are reported in Table 2. Significantly better visual acuities values were obtained for all distances with and without distance correction when compared with the values reported in the literature for a 12-month follow-up plus the established margins, of either 0.1 or 0.02 logMAR. The mean binocular uncorrected visual acuities were 0.01 ± 0.09 logMAR for far, 0.02 ± 0.08 logMAR for intermediate, and 0.04 ± 0.09 logMAR for near. The median and interquartile range for binocular uncorrected vision at the three distances was 0 and 0.1 logMAR, respectively. Figure 1A–C shows the monocular efficacy standard plots. The mean postoperative spherical equivalent (SE) was −0.24 ± 0.34 D (−0.25 [0.50] D). Figure 1D shows the percentage of eyes (68.92%) with an SE within ±0.25 D and those with an SE within ± 0.50 D (93.24%). All eyes achieved a postoperative SE within ±1.00 D. All eyes showed a refractive cylinder below 1.00 D for astigmatism (Figure 1E).

### 3.3. Contrast Sensitivity Defocus Curve and Light Distortion

The mean monocular contrast sensitivity defocus curve with a 95% confidence interval is shown in Figure 1F. From the biometric eye parameters measured and summarized in Table 1, only corneal spherical aberration for the mesopic pupil size was significantly related with the AUCs (T, rho = −0.44, *p* = 0.007; F, rho = −0.4, *p* = 0.01; I, rho = −0.42, *p* = 0.009; N, rho = −0.38, *p* = 0.02).

LDI was 18.82 ± 7.25% (19.42 (11.34)) for monocular vision and 15.64 ± 8.41% (14.64 (10.39)) for binocular vision. BFCr was 34.79 ± 6.89 (36 (11.30)) mm for monocular vision and 30.98 ± 6.35 (31.30 (11.35)) mm for binocular vision. Finally, BFCi was 0.44 ± 0.38 mm (0.37 (0.43)) for monocular vision and 0.45 ± 0.35 mm (0.43 [0.45]) for binocular vision. LDI was significantly correlated with spherical equivalent residual refraction, spherical aberration at mesopic pupil size, and the total area under the contrast sensitivity defocus curve. A multiple linear regression model significantly predicted the light distortion index with the sequential addition of SE (R2 = 0.19), TAUC (R2 = 0.37), and SA (R2 = 0.46) as prediction variables.

### 3.4. Patient Reported Outcomes

**Figure 2:**
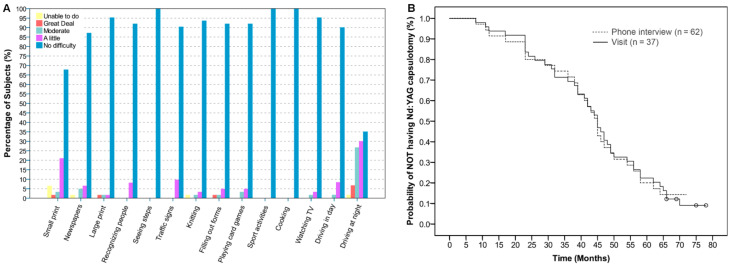
(**A**) Percentage of subjects cumulated for each answer of the VF-14 questionnaire (*n* = 62). (**B**) Survival plot for Nd:YAG rates.

Figure 2A shows the results of the VF-14 questionnaire. All of the questions were answered except for two subjects who did not answer the questions related to driving as they did not drive. No difficulty was found in more than 85% of subjects for all of the tasks, except for small print reading and driving at night. The total score was 94.73 ± 6.8 (96.43 (4.46)). Table 3 shows the results for the PRSIQ and for the additional questions.

No significant correlations of any of the light distortion analysis variables were found for either of the questions related to visual function during night vision driving or disturbances of photic phenomena during night vision (*p* > 0.05). Significant associations were found between the decision to be implanted with the same MIOL and satisfaction with vision at far (d = 0.38, *p* = 0.01), intermediate (d = 0.51, *p* = 0.001), and near distance (d = 0.40, *p* = 0.002), but not with photic phenomena disturbances (d = −0.11, *p* = 0.25).

### 3.5. Safety

No secondary surgeries were required. No other adverse events beyond those described in the previous recruitment section were identified either during the interview or in the study visit.

From the 62 patients interviewed, 89 (71.77%) eyes required Nd:YAG (Alcon YAG laser 3000 LE Nd) capsulotomy during the 6-year follow-up. A total of 11 (17.7%) subjects did not require Nd-YAG capsulotomy in any of both eyes, whereas 13 subjects (21%) required this procedure in one eye and 38 (61.3%) in both eyes. A stratified analysis was conducted for the 37 subjects attending the study visit. According to this, 55 eyes (74%) from 31 subjects (84%) needed Nd:YAG capsulotomy during the 6-year follow-up as surgeon criteria, due to a decrease in visual performance. From the 19 eyes (26%) without previous Nd:YAG capsulotomy, nine (12%) were classified as zero level of PCO density, whereas eight (11%) and two (3%) were classified as grades 1 and 2, respectively. Non-significant differences were obtained between the survival analysis from the call interview data and from the stratified analysis (*p* > 0.05); therefore, the data obtained in the study visit was used for a better interpretation (Figure 2B). The median time to require Nd:YAG capsulotomy obtained from the survival plot was 45 months (95% CI, 41.56 to 48.44 months). The mean time for the described procedure was 43.97 ± 48.27 months. The anterior capsular opacification was graded as zero for 72 eyes (97%) and as grade 1 for 2 the eyes of 2 subjects (3%).

## 4. Discussion

Although long-term studies with follow-up periods above 5 years have been reported in the past for some bifocal intraocular lenses [23,24], there are no long-term studies reported to date with such a long follow-up period for trifocal MIOLs. The main aim of this study was to report the efficacy results of a selection of interviewed subjects implanted with a specific model of diffractive trifocal IOL in our center from March 2014 to June 2015 (6-year follow-up). For this purpose, patient reported outcomes were collected for all patients who were able to be contacted by a phone call interview, and patients were consecutively invited for a visual performance assessment to our center, including the use of new metrics, such as contrast sensitivity defocus curves and light distortion analysis.

The efficacy results in the 37 eyes of the study evaluated showed non-inferiority in comparison with the previously reported 12-month results [6]. Indeed, the results of the present study were better than those reported by Mojzis et al. [6] for all efficacy variables, except for UDVA. The reason for the superiority of our outcomes in comparison with Mojzis et al. [6] may be that their sample at 12 months after surgery showed higher PCO rates with a lower number of Nd:YAG capsulotomies performed (3% in the present study with significant PCO during time of exploration versus the 15.8% reported in Mojzis et al. [6] study). Certainly, the efficacy results of the current series were more consistent with those reported in studies with shorter periods of time. A mean 6-year postoperative CDVA of −0.05 logMAR was found in the current study, which is a similar value compared to those reported in other studies at 3 and 6 months after implantation of the 839MP IOL (between −0.01 and −0.05 logMAR) [3,25,26]. The mean DCIVA in the present study was 0.08 logMAR, which is similar to the mean values of 0.06 logMAR and 0.07 logMAR reported for 3- and 6-month follow-ups [3,25,26]. Similarly, the mean DCNVA was 0.05 logMAR, which is similar to the mean values of 0.07 logMAR and 0.06 logMAR also reported for the 3- and 6-month follow-ups [25,26].

Some new metrics were also included in the current study. Particularly, CSDC has not been reported for short periods of time with the 839MP IOL. Comparing the performance for 0 D defocus location, the point that usually shows less difference in MTF between trifocal IOLs [27], the level slightly above 0.8 logCS found for a background brightness of 85 cd/m^2^ in the current series was very close to the mean of 0.83 logCS obtained with several MIOLs for a background brightness of 250 cd/m^2^ [28]. This suggest that not only visual acuity remains stable 6 years after surgery, but also probably contrast sensitivity.

Another new metric that has been already measured with the 839MP IOL, but in short term periods, is the level of light distortion. Brito et al. [5] reported a monocular LDI, BFCr, and BFCi of 46.97%, 55.28 mm, and 5.71 mm, respectively. The results in the current series were considerably lower, with means of 18.82%, 34.79 mm, and 0.44 mm, respectively, for the same variables. According to this, it can be hypothesized that light distortion decreases with time, but it seems difficult to believe that the long-term results with the 839MP IOL could be lower than the short-term data reported by Brito et al. [5] with a monofocal IOL (i.e., 23.94% for the LDI). More studies are needed to confirm if this potential trend in a reduction in light distortion parameters may be related to neuroadaptation, considering that some changes in brain visual processing have been found to be associated to neuroadaptation to multifocality [29]. LDI was also related to some other biometric parameters, decreasing with the increase of the spherical equivalent and with the increase of the total area under the CSDC. LDI was measured with best correction at infinity with addition of a +0.50 D lens; therefore, this increase in LDI can be only explained by aberrations induced by cumulating lenses in the trial frame. The correlation between the LDI and sphere was also reported by Brito et al. [5]. Furthermore, the correlation of LDI with CSDC confirmed that patients with a higher optical quality also had less LDI. This inverse relationship between contrast sensitivity and LDI was also reported by Escandón-García et al. [30]. LDI was also related with the increase of positive spherical aberration for a mesopic pupil size, which agrees with the results reported by Macedo-de-Araújo et al. [31].

The average total score for the VF-14 questionnaire was 94.73, a value close to the mean score of 93.58 reported by Liu et al. [26]. Total spectacle independence was achieved at far and intermediate distance vision, but 19.4% of subjects reported the need for spectacle use at a near distance, 6.5% reported this sometimes, and 12.9% seldom. The results of the current study were more optimistic than those reported by Mendicute et al. [2], and equal than those reported by Kohnen et al. [4] and Vargas et al. [32] for far and intermediate distance vision. However, these three studies reported slightly poorer levels of spectacle independence at a near distance compared with the present study, with 10.8%, 12%, and 10% of subjects, respectively, reporting the use of spectacles at near distances occasionally. Considering all of the interviewed patients, high satisfaction rates were obtained, with more than 90% of them being satisfied or very satisfied at all distances, as previously reported by Liu et al. [26] (general satisfaction), but obtaining better results than that of Mendicute et al. [2] (>80% at intermediate and near) and poorer than those reported by Vargas et al. (100%) [32]. The high satisfaction rates explained that 88.9% of subjects would have taken the decision to be operated on again following the same procedure, close to the 92% rate reported by Kohnen et al. [4] and lower than the 98% rate reported by Mendicute et al. [2].

No explants were conducted in any of the implanted eyes, but four (4.35%) subjects were excluded due to eye diseases that had developed in the course after surgery up to the 6 years of follow-up, and four subjects (4.35%) required corneal laser refractive retreatment. This retreatment rate was lower than the 10.8% reported by Gundersen et al. [33] which is explained by the low residual astigmatism found in our sample, with all eyes with a residual astigmatism of 1.00 D or below. Indeed, these authors indicated that residual astigmatism was one of the main reasons for retreatment in their sample. The most frequent device-related ocular adverse event was PCO, which required Nd:YAG treatment in 71.77% of the implanted eyes in a median time of 45 months. Bilbao-Calabuig et al. [10] reported a PCO rate with the 839MP IOL of 35% during 34 to 44 months. In the current series, the PCO rate for this same period of follow-up was slightly less than 30%. In any case, considering the higher increase in the slope of the survival plot after 40 months in the current study, both studies would probably have converged in similar PCO rates at 6 years. The question has recently arisen whether Nd:YAG laser rate is expected to be risen in the future due to major affectation of mild PCO with MIOL than monofocal IOLs [34]. The results from our study were in agreement with this hypothesis, but efficacy and satisfaction were maintained in the long-term and no adverse events secondary to Nd:YAG laser were reported in any on the reviewed or interviewed patients.

Our study has some limitations. Firstly, from the 92 patients implanted, 15 were not able to be contacted with the phone number retrieved in the clinical history; therefore the PROs did not contain a representation of all of the operated patients. A second limitation is related to assess Nd-YAG rates retrospectively or asking during the interview phone call, as the PCO grade could not be evaluated in the long term for these patients and some loss of information might have taken place. However, this was the reason we separately presented Nd-YAG rates for eyes attending the study visit and for those accounted through the clinical history or by phone, showing very similar lines in the survival plot. Finally, the cross-sectional study design led us to be cautious interpreting some findings, such as the possible reduction of light distortion with time.

## 5. Conclusions

In conclusion, this study reports the results for the longest follow-up time to date (6-years) after the implantation of a trifocal intraocular lens (AT LISA tri 839MP). Efficacy was equal or better that the reported in previous literature at 12-months. PCO treatment with Nd:YAG capsulotomy was generally required, but no adverse events secondary to Nd:YAG treatment were found. PRO results in terms of little difficulties conducting daily tasks or high satisfaction rates were comparable to those reported in short term studies.

## Figures and Tables

**Table 1:** Demographic and biometric characteristics of the sample recruited for study visit (*n* = 37).

	Mean ± SD	Median [IQR]
Age at follow-up visit	61.03 ± 5.47	61 [8.50]
IOL Master 500		
Axial length (mm)	23.33 ± 1.14	23.15 [1.51]
Mean corneal anterior keratometry (D)	43.70 ± 1.37	43.95 [1.64]
Pentacam AXL		
Anterior lens position (mm)	4.49 ± 0.35	4.45 [0.39]
Intraocular lens power (D)	21.41 ± 3.18	22 [4.50]
Irregular astigmatism at 4 mm (µm)	0.15 ± 0.05	0.15 [0.09]
Regular astigmatism (D)	0.49 ± 0.27	0.40 [0.45]
Corneal spherical aberration for mesopic pupil size (µm)	0.17 ± 0.13	0.14 [0.18]
Keratograph 5M		
Photopic pupil diameter (mm)	2.96 ± 0.49	2.90 [0.70]
Mesopic pupil diameter (mm)	5.17 ± 0.85	5 [1.20]

SD: Standard deviation; IQR: Interquartile range.

**Table 2:** Efficacy at 6-years for the random eye in comparison with that reported 12-months postoperatively in previous literature.

	6-Year Follow-up Mean ± SD Median [IQR]	12-Month Median from Literature	z-Test, *p*-Value Margin of 0.1 logMAR	z-Test, *p*-Value Margin of 0.02 logMAR
UDVA	0.06 ± 0.12 0.1 [0.1]	0.02	−1.75, 0.04	2.001, 0.023
CDVA	−0.05 ± 0.07 −0.1 [0.1]	0.02	−5.18, <0.0005	−4.53, <0.0005
UIVA	0.07 ± 0.09 0.0 [0.1]	0.12	−4.84, <0.0005	−3.31, <0.0005
DCIVA	0.08 ± 0.09 0.1 [0.1]	0.12	−4.85, <0.0005	−3.50, <0.0005
UNVA	0.08 ± 0.10 0.1 [0.1]	0.22	−5.33, <0.0005	−5.05, <0.0005
DCNVA	0.05 ± 0.06 0 [0.1]	0.22	−5.44, <0.0005	−5.44, <0.0005

UDVA—uncorrected distance visual acuity; CDVA—corrected distance visual acuity; UIVA—uncorrected intermediate visual acuity; DCIVA—distance corrected intermediate visual acuity; UNVA—uncorrected near visual acuity; DCNVA—distance corrected near visual acuity.

**Table 3:** Descriptive results for the patient reported spectacle independence questionnaire (PRSIQ) and the additional questions. Centrality and variation indices are in the first column and percentage of answers for each category (frequency) are in the Cat. columns.

PRSIQ	Mean ± SD; Median [IQR]	Cat. 1	Cat. 2	Cat. 3	Cat. 4	Cat. 5
Need Glasses						
Distance	2 ± 0; 2 [0]	0	100			
Intermediate	2 ± 0; 2 [0]	0	100			
Near	1.81 ± 0.40; 2 [0]	19.4	80.6			
Often wear						
Distance	5 ± 0; 5 [0]	0	0	0	0	100
Intermediate	5 ± 0; 5 [0]	0	0	0	0	100
Near	4.74 ± 0.57; 5 [0]	0	0	6.5	12.9	80.6
Comfortably without wear						
Distance	1.06 ± 0.36; 1 [0]	96.8	0	3.2	0	0
Intermediate	1.06 ± 0.36; 1 [0]	96.8	0	3.2	0	0
Near	1.37 ± 0.83; 1 [0]	77.4	16.1	6.5	0	0
Coding: “Yes” (Category 1) and “No” (Category 2). Wear and function items used verbal response labels of “all of the time” (Category 1), “most of the time” (Category 2), “Some of the time” (Category 3), “A little of the time” (Category 4), and “None of the time” (Category 5).						
Additional Questions (X)						
Satisfaction (satisfied)						
Distance	4.63 ± 0.75; 5 [1]	0	4.8	1.6	19.4	74.2
Intermediate	4.56 ± 0.88; 5 [1]	1.6	4.8	1.6	19.4	72.6
Near	4.50 ± 0.88; 5 [1]	0	8.1	1.6	22.6	67.7
Photic phenomena (bothersome)	2.21 ± 1.29; 2 [1]	32.3	45.2	3.2	8.1	11.3
Operated again (likely)	4.53 ± 0.95; 5 [1]	1.6	6.5	3.2	14.5	74.2
Coding for main words (X = satisfied or bothersome or likely): “not at all X” (Cat. 1), “slightly X” (Cat. 2), “neutral” (Cat. 3), “X” (Cat. 4), and “very X” (Cat. 5).						
